# The Activity of Natural Polymorphic Variants of Human DNA Polymerase β Having an Amino Acid Substitution in the Transferase Domain

**DOI:** 10.3390/cells12091300

**Published:** 2023-05-02

**Authors:** Olga A. Kladova, Timofey E. Tyugashev, Elena S. Mikushina, Nikita A. Kuznetsov, Daria S. Novopashina, Aleksandra A. Kuznetsova

**Affiliations:** 1 Institute of Chemical Biology and Fundamental Medicine, Siberian Branch of Russian Academy of Sciences, Novosibirsk 630090, Russianikita.kuznetsov@niboch.nsc.ru (N.A.K.);; 2 Department of Natural Sciences, Novosibirsk State University, Novosibirsk 630090, Russia

**Keywords:** DNA repair, DNA polymerase beta, single-nucleotide polymorphism, enzymatic activity

## Abstract

To maintain the integrity of the genome, there is a set of enzymatic systems, one of which is base excision repair (BER), which includes sequential action of DNA glycosylases, apurinic/apyrimidinic endonucleases, DNA polymerases, and DNA ligases. Normally, BER works efficiently, but the enzymes themselves (whose primary function is the recognition and removal of damaged bases) are subject to amino acid substitutions owing to natural single-nucleotide polymorphisms (SNPs). One of the enzymes in BER is DNA polymerase β (Polβ), whose function is to fill gaps in DNA with complementary dNMPs. It is known that many SNPs can cause an amino acid substitution in this enzyme and a significant decrease in the enzymatic activity. In this study, the activity of four natural variants of Polβ, containing substitution E154A, G189D, M236T, or R254I in the transferase domain, was analyzed using molecular dynamics simulations and pre-steady-state kinetic analyses. It was shown that all tested substitutions lead to a significant reduction in the ability to form a complex with DNA and with incoming dNTP. The G189D substitution also diminished Polβ catalytic activity. Thus, a decrease in the activity of studied mutant forms may be associated with an increased risk of damage to the genome.

## 1. Introduction

The occurrence of mutations in the genome is an unavoidable consequence of oxidative stress, deamination, alkylation, formation of apurinic/apyrimidinic (AP) sites, and single- or double-strand breaks in DNA [1,2,3,4,5,6]. Various repair systems exist to maintain the original DNA sequence [7,8,9,10]. One such system is base excision repair (BER), which recognizes predominantly non-bulky damage to heterocyclic bases and AP sites [7]. This system includes several enzymes: DNA glycosylases, which hydrolyze the *N*-glycosidic bond and remove the damaged DNA base; AP endonuclease incising the AP site, which may remain after the action of monofunctional DNA glycosylases; DNA polymerases, which fill the formed gap with complementary dNMPs; and DNA ligase, which repairs DNA bonds [8,10,11,12,13,14,15,16]. Normally, the BER pathway works efficiently, ensuring a high stability of genetic information. Nonetheless, genes of the repair enzymes themselves can undergo mutations. The most common type of mutations is single-nucleotide variations (SNVs), some of which can result in a change of an amino acid residue in the protein encoded by the affected gene [17]. If such SNV occurs in the population more often than 1%, then it is called single nucleotide polymorphisms (SNP). Numerous known SNPs and protein mutants have been described in the literature [18,19,20,21]. One of the cases where SNPs can have the most serious effect on the functioning of the whole BER pathway is mutations in the gene of DNA polymerase β (Polβ) because this enzyme must ensure rapid and accurate incorporation of a new dNTP for subsequent restoration of the original DNA sequence. Therefore, any disturbance of its natural activities can raise the frequency of mutations in the genome.

To date, many polymorphic variants with reduced enzymatic characteristics have been described for DNA polymerase β [22,23,24,25,26]. Moreover, up to 30% of analyzed human tumors have been shown to express Polβ mutants [27]. Using online software, the effect of an SNV can be successfully predicted that results in a substitution in an original amino acid residue and change a class of an amino acid residue [28]. In that report, an analysis of almost 200 Polβ variants revealed that 72 mutations (more than 36% of the total) are located in the DNA transferase domain, containing highly conserved residues Asp190, Asp192, and Asp256, which coordinate Mg^2+^ ions and are necessary for catalysis of nucleotide incorporation. Among the analyzed SNV-associated amino acid substitutions in the transferase domain, some had the greatest predicted negative effect on the functioning of Polβ: E154A (rs780232014), G189D (rs1331779238), M236T (rs149882093), and R254I (rs756393596) [28].

**Figure 1:**
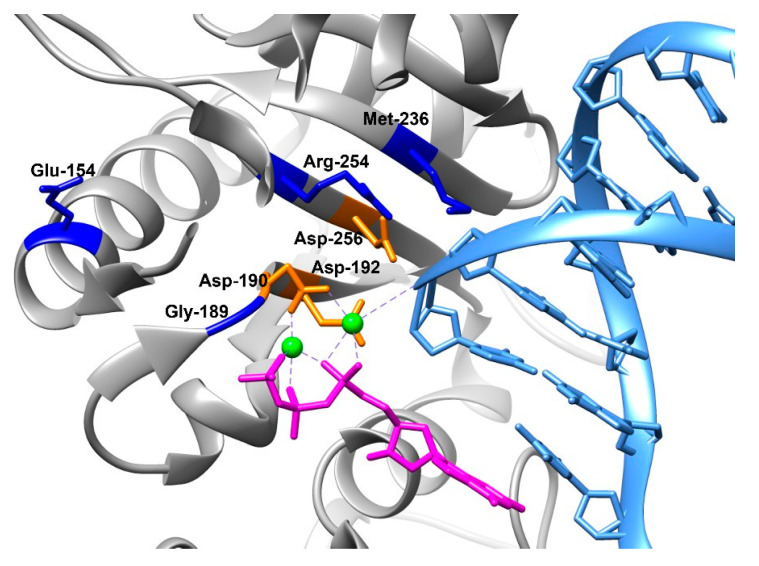
Crystal structure of human Polβ (grey) complexed with DNA containing a 1 nt gap (light blue) and with incoming nonhydrolyzable dGTP (magenta) (Protein Data Bank [PDB] ID 4PHE). The catalytic aspartate residues (orange) are shown, as are Mg^2+^ ions (green) and selected SNP-affected amino acid residues: Glu-154, Gly-189, Met-236, and Gly-254 (blue).

Analysis of crystal structure of human Polβ complexed with DNA and incoming dGTP has revealed that Glu-154 is located in an α-helix, away from bound DNA and incoming dNTP (Figure 1). Therefore, Glu-154 does not come into any direct contact with catalytic amino acid residues, DNA, or dNTP. The location of Gly-189 is the nearest to catalytic Asp-190; moreover, backbone atoms of Gly-189 coordinate the γ-phosphate of the incoming nucleotide [29]. Of note, mutant G189V has been found in liver cancer (https://hive.biochemistry.gwu.edu/biomuta, accessed on 20 February 2021), and Gly-189 is a part of conserved sequence Gly-Asp-Met-Asp in Polβ [30]. At the same time, the G189A substitution did not affect the level of dCTP incorporation when polymerase activity was tested in crude *Escherichia coli* extracts carrying the pTMbβ plasmid [30]. Met-236 is located in the DNA-binding site. The M236I variant has been detected in liver cancer (https://hive.biochemistry.gwu.edu/biomuta, accessed on 20 February 2021), supporting the assumption that the emergence of bulky aliphatic amino acid substitutions in the active site can disrupt its optimal geometry necessary for a catalytically competent state. Arg-254 is also situated near catalytic Asp-256, close to the DNA- and dNTP-binding sites. It is known that some mutations at this position lead to a decrease in the catalytic activity of the enzyme and its affinity to DNA [31]. The R254I variant has been found in uterine cancer (https://www.cbioportal.org and https://hive.biochemistry.gwu.edu/biomuta, accessed on 20 February 2021), indicating a possible negative impact of this mutation. 

In the present study, we performed molecular dynamics (MD) simulations and pre-steady-state kinetic analysis of four SNP-associated amino acid substitutions in the transferase domain of human Polβ, namely E154A, G189D, M236T, and R254I, to evaluate the efficiency of polymerase activity of these four natural variants of enzyme.

## 2. Materials and Methods

### 2.1. Site-Directed Mutagenesis and Protein Purification

Substitutions E154A, G189D, M236T, and R254I within the Polβ coding sequence were generated by site-directed mutagenesis. Primer sequences are presented in Table 1. The recombinant proteins were expressed in the *E. coli* Rosetta 2 strain carrying the pET-28c plasmid with a mutated Polβ sequence. The cultivation was conducted at 37 °C in 1.5 L of the Luria-Bertani (LB) medium supplemented with kanamycin (50 μg/mL) until optical density at 600 nm (OD_600_) reached 0.5–0.6; then, the temperature was lowered to 25 °C. Isopropyl-β-D-1-thiogalactopyranoside (IPTG) was added to a final concentration of 0.1 mM, after which expression was continued overnight at 25 °C. The cells were centrifuged and resuspended in 25 mL of Lysis Buffer (20 mM HEPES-KOH, 40 mM NaCl, pH 7.8). The 250 μL of a protease inhibitor cocktail was added to the cells, and the suspension was double lysed using a French press. The lysate was centrifuged at 40,000× *g* for 40 min. The supernatant was loaded onto a Q-Sepharose column pre-equilibrated with a buffer consisting of 20 mM HEPES-KOH pH 7.8 and 200 mM NaCl. The fraction containing protein was collected, supplemented with 500 mM NaCl and 15 mM imidazole, added to Ni-NTA resin, and incubated with stirring for 1.5 h at 4 °C. The Ni-NTA resin was washed with 10 mL of a buffer composed of 20 mM HEPES-KOH pH 7.8, 90 mM imidazole, and 500 mM NaCl to eliminate nonspecific protein binding. Then, the Ni-NTA resin was incubated for 5 min with 7 mL of a buffer consisting of 20 mM HEPES-KOH pH 7.8, 440 mM imidazole, and 500 mM NaCl to allow the bound protein to dissociate from the resin. The collected protein fraction was transferred to a dialysis bag and dialyzed overnight against a buffer composed of 20% of glycerol, 20 mM HEPES-KOH pH 7.8, and 150 mM NaCl. The pure protein fraction was supplemented with 50% of glycerol and stored at −20 °C.

### 2.2. Oligodeoxyribonucleotides

Sequences of the 2ʹ-oligodeoxyribonucleotides used in the work are given in Table 2. DNA substrates that were 36 bp long and labeled with FAM at the 5′ end were obtained by mixing equimolar amounts of three DNA strands: FAM_Pol19, Pol_36_N, and Pol16. DNA substrates containing a 2-aPu residue were prepared by mixing equimolar amounts of Pol16, Pol19, and Pol36_N_aPu. The DNA substrates were annealed for 5 min at 93 °C and allowed to cool down to room temperature.

### 2.3. Circular Dichroism (CD) Spectroscopy

CD spectra were recorded on a Jasco J-600 spectropolarimeter (Jasco, Tokyo, Japan). The concentration of Polβ in the device cell was 1.0 μM. The experiments were carried out in 50 mM Tris-HCl pH 7.5, 50 mM KCl, 1.0 mM EDTA and 5.0 mM MgCl**_2_** buffer in quartz cells with 0.1 mm light path length. The spectra were acquired at bandwidth 1.0 nm and wavelength 190 to 260 nm at room temperature. The scans were accumulated and automatically averaged. To describe the spectra, we used an online tool for fitting and simulation of CD spectra of proteins (https://bestsel.elte.hu, accessed on 1 February 2023) [32].

### 2.4. Fluorescence Thermal Shift Assay

The melting temperature was measured using the Quant Studio 5 Real-Time PCR System (Applied Biosystems, Waltham, MA, USA), in PCR tubes. Each tube contained 20 μL of solution consisting of 50 μM protein, 50 mM Tris-HCl pH 7.5, 50 mM KCl, 1.0 mM EDTA and 5.0 mM MgCl_2_ buffer and 5X ProteOrange dye (Lumiprobe). The temperature was elevated steadily in 0.028 °C steps from 25.1 °C to 99.9 °C. The fluorescence of the ProteOrange dye was measured using excitation at 470 nm and emission at 558 nm. Measurements were taken in duplicate. The value of the melting temperature was calculated using the Boltzmann sigmoidal curve equation:

F = F_u_ + (F_b_ − F_u_)/[1 + exp(T_m_ − x/slope)] (1)

where F is ProteOrange fluorescence emission, x is temperature, F_u_ is baseline fluorescence at low temperature, F_b_ is maximal fluorescence at the high temperatures, slope describes the steepness of the curve, and Tm is the melting temperature of the protein.

### 2.5. Molecular Dynamics (MD) Simulations

Binary open-state Polβ–DNA complex and ternary closed-state Polβ–DNA–dNTP complex models were based on crystal structures of human Polβ–DNA [33,34,35], with bound DNA edited to match truncated experimental oligonucleotide sequences. Human Polβ apo-enzyme model was built using Modeller interfaced with Chimera based on rat Polβ apo-enzyme crystal structure [36,37,38]. Simulations were performed using GROMACS MD suite [39]. The protein and the DNA primer were parameterized with AMBER 14SB-OL15 force field [40,41,42,43]. Nucleoside triphosphate parameters were obtained following an established approach, with partial charges fitted using R.E.D. Server [44,45]. Magnesium ions were simulated as octahedral dummy models [46]. Flat-bottomed distance restraints were applied to heavy atoms involved in hydrogen bonds of terminal base pairs to prevent truncated DNA fraying. Nonbonded interactions cutoff was set at 1.0 nm, long-range electrostatic interaction was treated using the PME method [47,48]. System temperature and pressure were maintained using a Bussi thermostat and a Parrinello–Rahman barostat [49,50]. The structures were solvated and neutralized in a dodecahedral PBC box with TIP3P model water and 50 mM KCl JC ions [51,52]. Steepest-descent energy minimization was followed by 1 ns NVT and NPT equilibrations with solute heavy atoms restrained. Unrestrained molecular dynamics simulations were run in triplicate for 100 ns, with one trajectory for each model extended up to 300 ns. Trajectory processing was performed using the integrated GROMACS toolset. Images were generated in the open-source version of PyMOL Viewer.

### 2.6. DNA-Binding Analysis

Determination of the effect of the amino acid substitutions on the stage of Polβ variants’ binding to a DNA substrate was conducted using the electrophoretic mobility shift assay (EMSA) in a nondenaturing 10% polyacrylamide gel (75:1) in 0.5× TBE buffer at 200 V. The enzyme variants were serially diluted in a buffer consisting of 50 mM Tris-HCl pH 7.5, 50 mM KCl, 1 mM Na_2_EDTA, 1 mM DTT, 5 mM Mg^2+^, and 7% of glycerol. Next, 5 µL of 100 nM DNA—a substrate consisting of three chains, Pol19_FAM, Pol16, and Pol36_N—was added to the enzyme. The resulting 10 µL mixtures were incubated for 15 min at room temperature and applied to the prepared gel. The separation of the reaction products took place at a voltage of 200 V for 30 min. After that, the gel was scanned using a VersaDoc gel documentation system, and data on the formation of the enzyme–substrate complex were processed in Gel-Pro 4.0. Dissociation constant *K*_d_ of each Polβ–DNA complex was calculated according to the equation:

Fraction bound (%) = F_u_ + (F_b_ − F_u_)/(1 + (*K*_d_/[Polβ])^h^) (2)

where h is Hill’s coefficient, F_u_ denotes a background’s contribution, and F_b_ represents maximal intensity of the complex.

### 2.7. The Polymerase Reaction Assay

To evaluate the activity of the four Polβ variants, a solution of a 1-nt-gapped DNA substrate (0.5 µM) and 5 µM complementary dNTP was mixed with a 0.5 µM enzyme solution. The reaction was carried out at 37 °C in a buffer composed of 50 mM Tris-HCl pH 7.5, 50 mM KCl, 1 mM EDTA, 5 mM MgCl_2_, 1 mM DTT, and 7% of glycerol. The enzymatic reaction was stopped with the addition of an equal volume of a stop solution (7.5 M urea, 25 mM EDTA, 0.1% of xylene cyanole, and 0.1% of bromophenol blue). The reaction products were separated in a denaturing 15% polyacrylamide gel. The resulting gel was visualized with the help of the VersaDoc gel-documenting system (Bio-Rad Laboratories, Hercules, CA, USA). The degree of substrate transformation was computed as the ratio of peak areas of the product to the sum of peak areas of the product and of the peak of the initial substrate in the Gel-Pro 4 analyzer software (Media Cybernetics, Rockville, MD, USA). The obtained data were fitted to the equation:

[Product] = A × (1 − exp(−*k*_obs_ × t)) (3)

where A is amplitude, *k*_obs_ is the rate constant, and t is reaction time.

### 2.8. Registration of DNA Conformational Changes

This analysis during the interaction of a Polβ variant and one of 2′-deoxyribonucleoside triphosphates was conducted using an SX.20 MV stopped-flow spectrometer (Applied Photophysics, Leatherhead, UK). Conformational alterations were registered by monitoring the changes of fluorescence intensity of 2-aPu residue. The excitation wavelength was λ_ex_ = 310 nm, and the emission wavelength λ_em_ = 370 nm. Concentrations of the reagents after mixing were 1 μM enzyme, 0.5 μM DNA substrate, and various concentrations of one of 2′-deoxyribonucleoside triphosphates. The reaction was allowed to proceed at 37 °C in a buffer consisting of 50 mM Tris-HCl pH 7.5, 50 mM KCl, 1 mM Na_2_EDTA, 1 mM DTT, 5 mM Mg^2+^, and 7% of glycerol.

To determine the rate constant of the polymerization reaction *k*_pol_ and observed constant of dissociation *K*_d,app(dATP)_ of 2′-deoxyribonucleoside triphosphate from the enzyme–DNA complex, the obtained data on the accumulation of the reaction product were processed via the equation:

F = F_0_ + F_1_ × exp(−*k*_obs_ × t) (4)

where F is observed 2-aPu fluorescence intensity, F_0_ is background fluorescence, F_1_ represents a fluorescence parameter, and *k*_obs_ denotes the observed rate constant.

A graph of the dependence of the observed rate constants on the dATP concentration was built to estimate the catalytic rate constant, *k*_pol_, and the apparent dissociation constant of dATP (*K*_d,app(dATP)_) via fitting to this equation:

*k*_obs_ = *k*_pol_ × [dATP]/(*K*_d, app_ + [dATP]) (5)

## 3. Results and Discussion

### 3.1. Effect of Substitutions on Protein Structure

**Figure 2:**
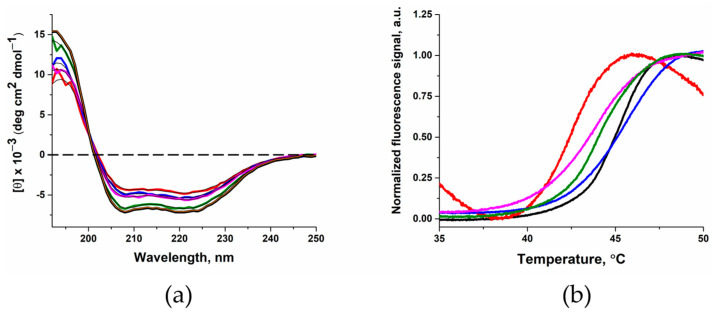
(**a**) CD spectra of WT Polβ (black) and of its variants E154A (red), G189D (green), M236T (blue), and R254I (magenta). The results of fitting are shown as solid lines: orange for WT Polβ and black for the variants. (**b**) Representative thermal melt curves for WT Polβ (black) and of its variants E154A (red), G189D (green), M236T (blue), and R254I (magenta).

To determine the effect of substitutions on the structure of the enzyme, the CD spectra was recorded, and the melting temperature was calculated using the thermal shift assay. Examination of the effect of the amino acid substitutions on secondary structure of the enzyme revealed that circular dichroism (CD) spectra almost match between wild type (WT) Polβ and the G189D variant (Figure 2a). By contrast, differences were found between WT Polβ and variants E154A, M236T, and R254I. Using a web resource for analysis of CD spectra (https://bestsel.elte.hu, accessed on 1 February 2023) [53,54,55,56], the α-helix content of the Polβ variants was calculated and revealed a decrease in the α-helix content in variants E154A, M236T, and R254I when compared with the WT enzyme (Table 3). These data indicated that three of the four substitutions have some destabilizing influence on the protein’s structure. Measuring the thermal stability of enzymes revealed little differences for tested variants, except for the mutant form E154A. The T_m_ value for E154A variant was 2.5 °C lower, indicating less thermal stability (Figure 2b, Table 3).

**Figure 3:**
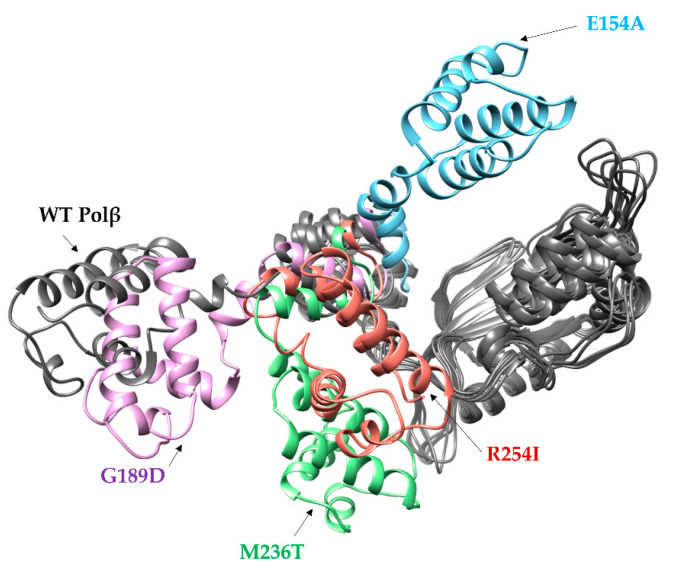
An overlay of representative snapshots of an unbiased MD simulation for human WT Polβ (grey) and its variants E154A (blue), G189D (purple), M236T (green), and R254I (red). The colored parts represent regions of the enzyme structure for which a position other than for the wild-type enzyme was observed. The part of a variant’s structure that overlaps with WT enzyme structure is also colored grey.

To reveal a possible reason for the protein structure destabilization by substitutions E154A, M236T, and R254I, structural models of all the Polβ variants under study were obtained using MD simulations. According to known apo enzyme Polβ structures [57,58,59], the enzyme is in a wide-open conformation if it is not bound to DNA or dNTP. In our MD data, it was found that the single-amino-acid substitution in E154A, M236T, and R254I, but not G189D, has a substantial impact on the arrangement of protein domains (Figure 3). Since the N-terminal region of the protein has a certain mobility, during the MD simulations different positions of the N-terminal region were observed for mutant forms. Variants E154A, M236T, and R254I adopt a more compact conformation as compared with the open-state conformation of the WT enzyme. Notably, in the WT enzyme, the Glu-154 amino acid residue forms a salt bridge with Arg-253, which normally takes part in the regulation of open and closed states of the enzyme. Therefore, a loss of this interaction in Polβ E154A leads to a disturbance of this regulation and stabilization of closed-state conformation. It is possible that substitutions M236T and R254I also disturb some inner contacts with other amino acid residues, thereby causing a more closed conformation of the enzyme as compared to the WT.

### 3.2. The DNA-Binding Ability of the Polβ SNP Variants

In an electrophoretic mobility shift assay (EMSA), the SNP variants were tested for their ability to bind DNA containing a 1 nt gap, with different nucleotides opposite the gap. Obtained dependences of the DNA-bound fraction (%) of the enzyme on the enzyme concentration allowed to calculate dissociation constant *K*_d_. Interesting to note that tested mutant variants had higher *K*_d_ value for Gap_G DNA than for other types of tested DNA substrates. The same observation was found recently for WT enzyme [26]. The exception was for M236T variant, which bound all types of DNA substrates with approximately 10-fold less affinity. 

**Figure 4:**
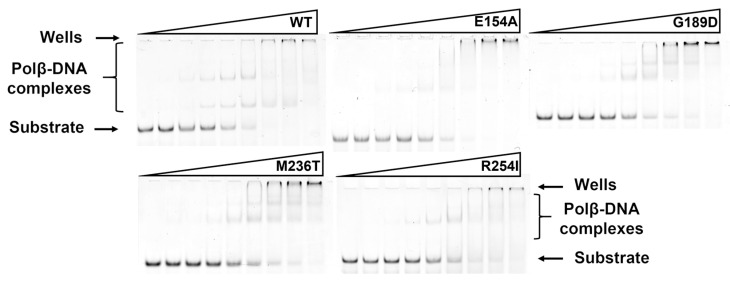
The EMSA of DNA binding by WT Polβ and by polymorphic variants. The experiments were conducted using serial dilutions of the enzymes. [Gap_C] = 50 nM.

**Figure 5:**
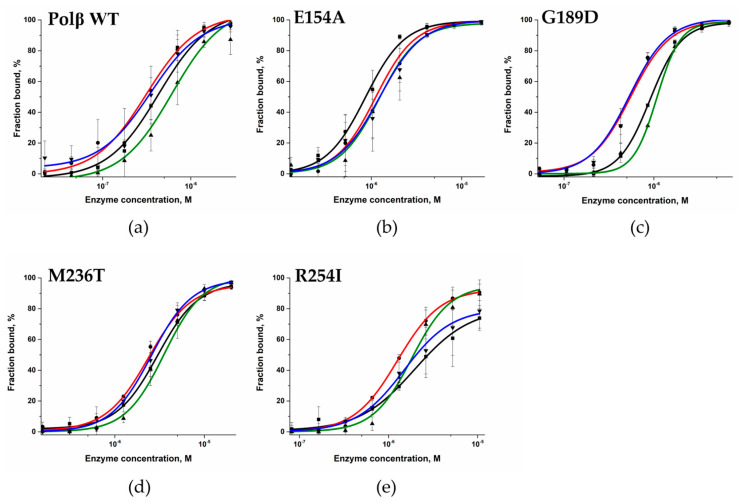
The DNA-binding ability of Polβ mutant variants as determined by the EMSA. The DNA-bound fraction (%) of the enzyme plotted against enzyme concentration: (**a**) WT Polβ (**b**) E154A, (**c**) G189D, (**d**) M236T, (**e**) R254I. The concentrations of WT Polβ were 22 nM to 2.63 μM, Polβ E154A were 0.129 to 16.5 μM, of G189D 53.4 nM to 6.83 μM, of M236T 0.156 to 20 μM, and of R254I 83 nM to 10.5 μM. The final DNA concentration was 50 nM. Black: Gap_A (■), red: Gap_T (●), green: Gap_G (▲), and blue: Gap_C (▼).

Overall, all tested SNP variants had higher dissociation constant *K*_d_ than WT Polβ did, indicating that the analyzed amino acid substitutions have a destabilizing impact on the Polβ–DNA binary complex (Figure 4 and Figure 5, Table 4).

**Figure 6:**
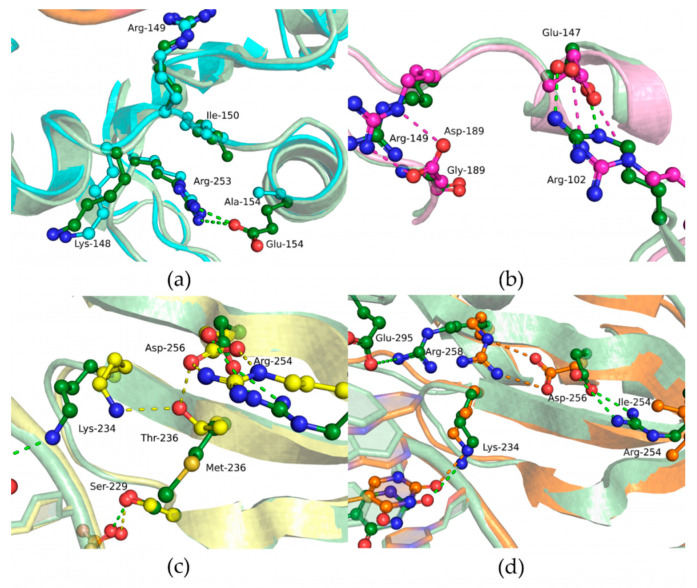
Close-up view of substitution-affected regions of WT Polβ (green) and of its variants (**a**) E154A (blue), (**b**) G189D (pink), (**c**) M236T (yellow), or (**d**) R254I (orange) in the modeled binary enzyme–DNA complex. Hydrogen bonds are presented as dashed lines.

Substitutions E154A and G189D showed a moderate negative impact on DNA binding (Table 4). The close-up view of the MD model of Polβ E154A revealed a loss of the salt bridge between amino acid residues Glu-154 and Arg-253 (Figure 6a), which influence the transition between open and closed states of the enzyme. Nevertheless, these data suggested that the maintenance of this contact is not important for DNA binding. In Polβ G189D, the Asp-189 residue seen in the dNTP-binding pocket forms a salt bridge with Arg-149; this event most likely should considerably affect dNTP binding but not DNA binding (Figure 6b).

The strongest effect on the DNA-binding ability was registered for substitution M236T. The binding affinity was approximately 10-fold weaker for this variant as compared to the WT enzyme. According to the MD simulations, when Met-236 is replaced by Thr, a hydrogen bond can form between Thr-236 and Lys-234, which in the WT enzyme engage in a hydrogen bond with a phosphate group of DNA (Figure 6c). Due to the relocation of the hydrogen bond to the Thr-236 residue, DNA coordination during the binding may be less effective, which is consistent with the findings in the EMSA.

The R254I variant also showed weaker affinity for the gapped DNA substrate for each possible DNA base opposite the gap (Table 4). In this context, the most destabilizing effect was registered for the DNA substrates containing a purine base opposite the gap. The substitution of Arg-254 by Ile induces a reorientation of Asp-256, giving rise to a salt bridge with Arg-258, and thereby results in this region’s stabilization, which is unfavorable for interaction with the Mg^2+^ ion and deprotonation of the 3′-OH end of DNA (Figure 6d). This interaction was found to not be advantageous in the WT enzyme, where Arg-258 forms salt bridges with Asp-192 or Glu-295.

### 3.3. DNA Polymerase Activity of Polβ SNP Variants

**Figure 7:**
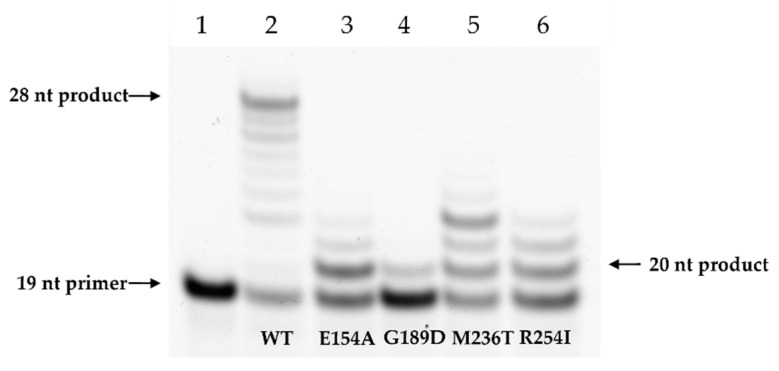
A comparison of DNA elongation efficiency in strand displacement synthesis by Polβ variants. Lane 1 corresponds to a 19 nt FAM-labeled DNA primer Gap_T, lane 2: elongation by WT Polβ, lane 3: by Polβ E154A, lane 4: by Polβ G189D, lane 5: by Polβ M236T, lane 6: by Polβ R254I. The greatest length of the product during the 1 min reaction was observed for the WT enzyme: a 28 nt product. The E154A variant elongated the primer by 3 nt, G189D by 1 nt, M236T by 5 nt, and R254I by 3 nt.

In the analysis of the ability of the Polβ variants to carry out strand displacement synthesis, it was observed that the tested SNP variants have a reduced ability to fill a 1 nt gap in DNA and to add a new nucleotide (Figure 7). Indeed, during a 1 min reaction, the WT enzyme catalyzed incorporation of 9 nt into the Gap_T DNA primer. Among the four evaluated SNP variants, M236T had the highest incorporation efficiency: an addition of no more than 5 nt to DNA. Polβ E154A and Polβ R254I had comparable efficiency and transferred only 3 nt. The slowest variant was G189D, which catalyzed the elongation inefficiently: only by a single nucleotide.

**Figure 8:**
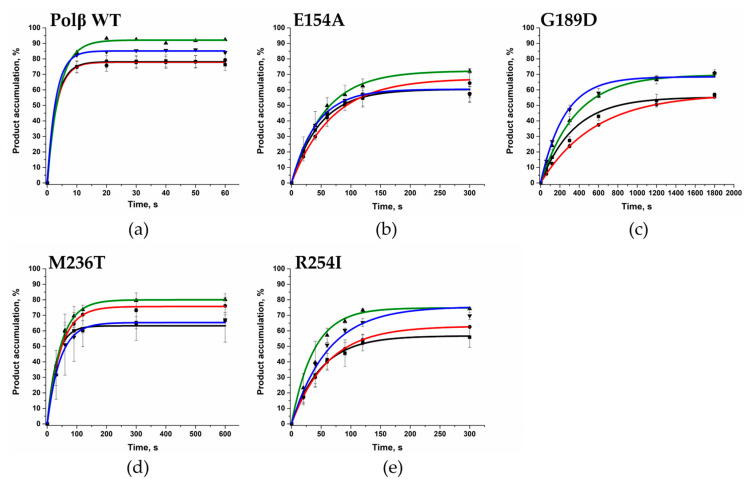
Single-nucleotide incorporation by the WT Polβ and polymorphic variants. (**a**) By WT Polβ, (**b**) by Polβ E154A, (**c**) by Polβ G189D, (**d**) by Polβ M236T, and (**e**) by Polβ R254I. Effects of the opposite nucleotide and of the nature of incoming dNTP on product accumulation are presented: Gap_A (■)/dTTP (black), Gap_T (●)/dATP (red), Gap_G (▲)/dCTP (green), and Gap_C (▼)/dGTP (blue). [DNA substrate] = 0.5 μM, [enzyme] = 0.5 μM, [dNTP] = 5 μM.

The enzymatic reaction in the presence of only one type of dNTP, which was complementary to nucleotide located opposite the gap, revealed that all the tested Polβ variants carry out the transferase reaction more slowly than WT Polβ does (Figure 8, Table 5). Overall, all variants (except G189D) had 10-fold lower *k*_obs_. It turned out that the activity of Polβ G189D was almost 100-fold lower than that of the WT enzyme.

### 3.4. dNTP Binding and Incorporation

**Figure 9:**
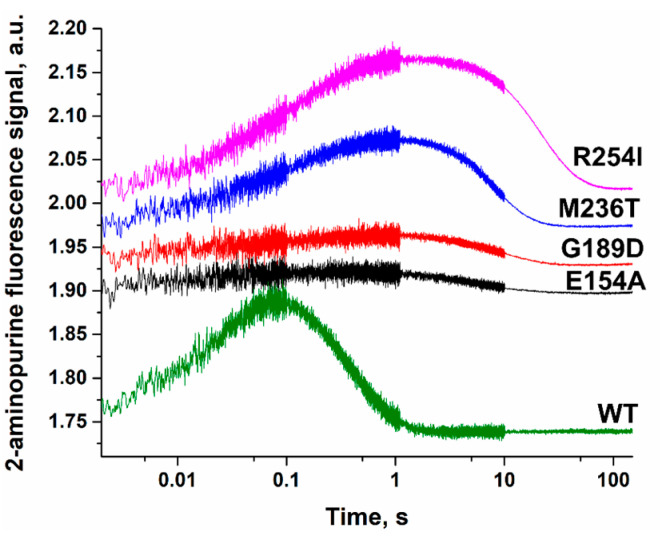
A comparison of changes of 2-aPu fluorescence intensity during the interaction of WT Polβ or its variants with Gap_T DNA and dATP. [DNA substrate] = 0.5 μM, [enzyme] = 1.0 μM, [dATP] = 100 μM.

For stepwise determination of the influence of the amino acid residue substitutions on the interaction with substrates, conformational changes in DNA were monitored with the stopped-flow technique during interaction with each Polβ variant. A DNA substrate containing a 2-aminopurine residue (2-aPu) as a fluorescent marker was used. During the dNTP binding and incorporation, a two-phase change in fluorescence intensity of 2-aPu was observed (Figure 9), as reported previously [26,60].

An increase in fluorescence intensity of 2-aPu denotes the assembly of a ternary complex of the enzyme with DNA and dNTP. For all the tested Polβ variants, this process seemed to proceed more slowly than for the WT enzyme. Furthermore, for variants E154A and G189D, the amplitude of the 2-aPu fluorescent signal was smaller. This finding suggested that for Polβ E154A, the loss of the salt bridge between Glu-154 and Arg-253 and a disturbance of the transition between open and closed states of the enzyme lead to inefficient DNA distortion in the ternary complex. On the other hand, for Polβ G189D, this effect is most likely associated with a reduced ability to bind incoming dATP and inefficient ternary-complex formation. Variants M236T and R254I showed the same amplitude of 2-aPu fluorescence signal as seen in the case of WT Polβ, but the rate of formation of the ternary complex was slower.

The next phase is a decrease in fluorescence intensity of 2-aPu and means the assembly of the catalytic complex and incorporation of the incoming nucleotide into the primer. For the WT enzyme, this process ended by time point 3 s, but all the variants showed a much slower decline of the 2-aPu fluorescent signal, indicating a reduced rate of product accumulation.

**Figure 10:**
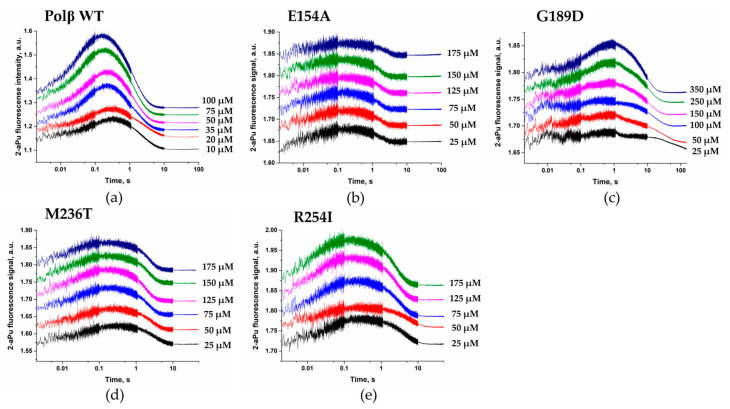
Kinetic curves of changes in 2-aPu fluorescence emission during the interaction of WT Polβ or its variants with DNA. (**a**) Polβ WT (**b**) E154A, (**c**) G189D, (**d**) M236T, and (**e**) R254I. The dATP concentration is indicated in the panels.

**Figure 11:**
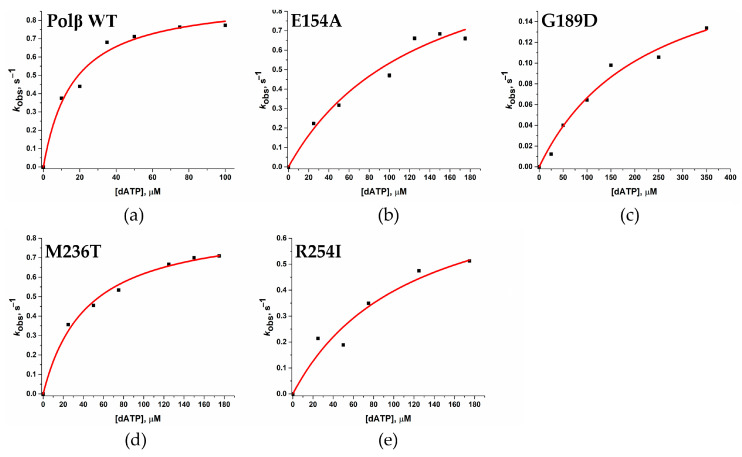
The dependence of calculated values of observed rate constants on the dATP concentration. (**a**) WT Polβ, (**b**) E154A, (**c**) G189D, (**d**) M236T, and (**e**) R254I.

To measure polymerization rate constant *k*_pol_ and apparent dissociation constant *K*_d,app (dNTP)_, a series of kinetic curves was obtained where various concentrations of dATP were applied (Figure 10). The section of the kinetic curves corresponding to the decrease in the 2-aPu fluorescence intensity was fitted to Equation (3) in order to calculate the observed rate constants *k*_obs_. Dependences of *k*_obs_ on the dATP concentration in the reaction for all studied variants of the enzyme (Figure 11) were fitted to Equation (4) and allowed to estimate (Table 6) rates of the chemical polymerization step (*k*_pol_) and the apparent dissociation constant *K*_d,app(dATP)_.

A comparison of the obtained rate constants of chemical polymerization step *k*_pol_ revealed that the G189D substitution leads to a four-fold slowdown, indicating a destabilizing effect of Asp-189 in the active site of this SNP variant. By contrast, no significant differences in polymerization constant *k*_pol_ were found between the WT enzyme and the other variants E154A, M236T, and R254I. This can mean that substitutions E154A, M236T, and R254I do not have a direct influence on the catalytic stage of Polβ.

The calculated apparent dissociation constant *K*_d,app (dATP)_ values revealed a destabilizing effect of each of the four substitutions. A 2.7-fold impairment of dATP binding was observed in the case of M236T, and an approximately eight-fold impairment was documented for variants E154A and R254I. The greatest effect of an amino acid substitution was seen in Polβ G189D, in which *K*_d,app (dATP)_ was 14-fold higher than that of WT Polβ.

**Figure 12:**
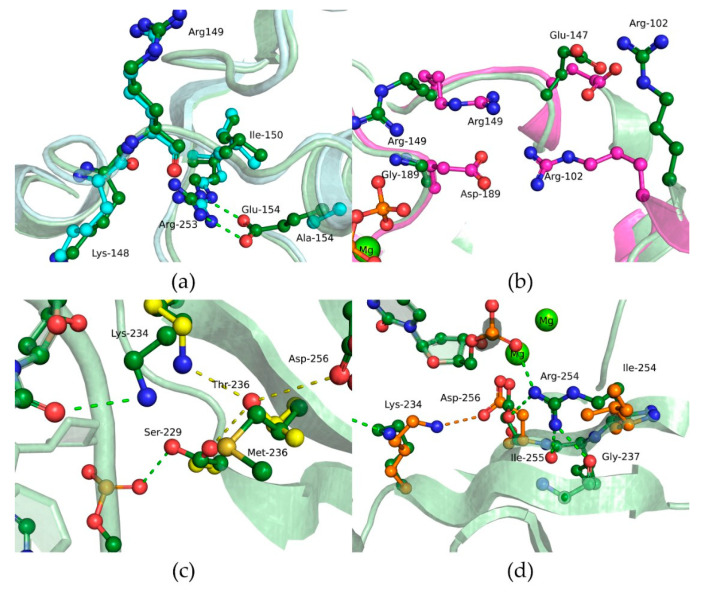
Close-up view of substitution-affected regions of WT Polβ (green) and of its variants (**a**) E154A (blue), (**b**) G189D (pink), (**c**) M236T (yellow), or (**d**) R254I (orange) in the modeled ternary complex enzyme–DNA–dNTP. Hydrogen bonds are depicted as dashed lines.

To elucidate the effect of each amino acid substitution on dNTP binding, MD simulations of ternary complexes of Polβ variants with DNA and dNTP were performed next (Figure 12). It was found that the E154A variant lacks the salt bridge between Glu-154 and Arg-253 (Figure 12a), as in the binary complex Polβ–DNA (Figure 6a). The importance of such inner stabilization was confirmed by the existence of structurally conserved bridges in this protein region within DNA polymerases of the X family. Indeed, this type of interaction involves different amino acid residues but is present in other enzymes; for example, in human and Arabidopsis DNA polymerases λ, it is completely preserved; Polβ of Leishmania and Dpo4 of *Saccharomyces cerevisiae* also have this salt bridge. In human DNA polymerase μ and terminal deoxynucleotidyl transferase TdT, the salt bridge is formed by Asp-294 and Lys-413 and by Glu-307 and Lys-429, respectively. *Thermus thermophilus* PolX has a charge-inverted salt bridge between Arg-158 and Asn-238, which apparently plays a similar structural role. Although in our analysis, direct contacts within the Glu-154 region that could influence the dNTP binding ability were not revealed, the E154A substitution had an obvious considerable effect on the interaction with dNTP.

The effect of substitution G189D was the most predictable because Gly-189 is located most closely to catalytic Asp-190 and because backbone atoms of Gly-189 coordinate the γ-phosphate of the incoming nucleotide (Figure 6b). An additional residue, Asp-189 bearing a negative charge induces an appreciable disturbance in the active site, thus distorting active-site geometry, influencing Mg^2+^ ion coordination, and strongly affecting dNTP binding. All these factors led to a lower rate constant of the chemical step and inefficient dATP binding (Table 6).

In the case of the M236T substitution, the impact on the chemical step and on the apparent dissociation constant was the weakest. MD simulation data revealed that the Thr-236 hydroxyl group in Polβ M236T can maintain hydrogen bonds with Ser-229, Lys-234, and Asp-256 (Figure 6c). Of note, human Polβ’s Met-236 residue corresponds to Thr-233 in *T. thermophilus* PolX. Moreover, it is reported [61] that the Met-236 residue appears to transmit fine structural perturbations to catalytic metal-coordinating residue Asp-256, thereby affecting its conformational stability. The activity of variants M236A and M236L is also slightly lower in comparison with the WT enzyme [61].

The R254I substitution caused a loss of contact with a DNA phosphate group and reorientation of the Asp-256 side chain thus giving rise to a salt bridge with Lys-234 (Figure 6d), which most likely disturbs dNTP binding. On the other hand, throughout the MD trajectory of the ternary complex, DNA was stable in a position similar to that in the complex with the WT enzyme.

## 4. Conclusions

Maintaining the Polβ activity at a normal level is essential because even slight deviations of its activity can affect the frequency of mutations in the genome. In this work, we tested the effect of four natural polymorphic variants of Polβ—containing substitution E154A, G189D, M236T, or R254I—on DNA binding, dNTP binding, and catalytic activity. These substitutions have previously been predicted to potentially have a strong negative effect on Polβ functions. All the tested variants were found here to have a weaker ability to fill single-nucleotide gaps in DNA substrates, with the G189D variant being the slowest. A stepwise analysis of the effect of the amino acid substitutions revealed that all the studied Polβ variants bind to DNA with weaker affinity than the WT enzyme does. The impact of the substitutions in question on the binding to dATP during the formation of a ternary complex was estimated using MD simulations and kinetic assays. It was found that this process is slower for all the Polβ variants than for the WT enzyme. It is noteworthy that only the G189D substitution directly affects the polymerization rate constant *k*_pol_, while the decrease in the activity of the other assessed Polβ variants apparently is not associated with a direct deterioration of the catalytic ability. Thus, our findings indicate that substitutions that do not even affect catalytically active amino acid residues or are located far away from the active site can substantially diminish the total enzyme efficiency.

## Figures and Tables

**Table 1:** Primers for site-directed mutagenesis.

Polβ Variant	Primer Sequences
E154A	Forward 5′-GGACTTTGAAAAAAGAATTCCTCGTGAAGCGATGTTACAAATGC-3′ Reverse 5′-GCATTTGTAACATCGCTTCACGAGGAATTCTTTTTTCAAAGTCC-3′
G189D	Forward 5′-GAGGTGCAGAGTCCAGTGATGACATGGATGTTCTCC-3′ Reverse 5′-GGAGAACATCCATGTCATCACTGGACTCTGCACCTC-3′
M236T	Forward 5′-GGGTGAGACAAAGTTCACGGGTGTTTGCCAGC-3′ Reverse 5′-GCTGGCAAACACCCGTGAACTTTGTCTCACCC-3′
R254I	Forward 5′-GAATATCCACACAGAATAATTGATATCAGGTTGATACCCAAAGATC-3′ Reverse 5′-GATCTTTGGGTATCAACCTGATATCAATTATTCTGTGTGGATATTC-3′

**Table 2:** Sequences of oligodeoxyribonucleotides used in the work.

Name	Sequence
Pol16	5′-TAGTCACCTCAATCCA-3′
Pol19	5′-GCCTCGCAGCGGTCCAACC-3′
Pol19_FAM	FAM 5′-GCCTCGCAGCGGTCCAACC-3′
Pol36_N_aPu	5′-TGGATTGAGGTGACTA(2-aPu)NGGTTGGACGGCTGCGAGGC-3′
Pol36_N:	5′-TGGATTGAGGTGACTANGGTTGGACGGCTGCGAGGC-3′

**Table 3:** The calculated α-helix content and T_m_ value for Polβ variants.

	WT	E154A	G189D	M236T	R254I
α-helixes, %	79 ± 16 *	41 ± 8	68 ± 14	32 ± 6	59 ± 12
T_m_, °C	44.9 ± 0.2	42.4 ± 0.1	45.2 ± 0.1	44.2 ± 0.8	44.1 ± 0.6

* Data from ref. [26].

**Table 4:** Dissociation constants *K*_d_ (μM) of the enzyme–DNA complex.

Substrate	WT *	E154A	G189D	M236T	R254I
Gap_A	0.38 ± 0.02	0.86 ± 0.04	0.97 ± 0.03	3.0 ± 0.1	2.0 ± 0.2
Gap_T	0.33 ± 0.03	1.1 ± 0.1	0.55 ± 0.05	2.6 ± 0.1	1.26 ± 0.04
Gap_G	0.59 ± 0.07	1.32 ± 0.04	1.26 ± 0.03	2.4 ± 0.1	1.8 ± 0.2
Gap_C	0.38 ± 0.03	1.2 ± 0.1	0.53 ± 0.04	3.5 ± 0.3	1.5 ± 0.2

* Data from ref. [26].

**Table 5:** Observed rate constants *k*_obs_ (s^−1^) of gap-filling incorporation of various dNTPs into the DNA.

*k*_obs_, s^−1^	WT *	E154A	G189D	M236T	R254I
Gap_A	0.33 ± 0.03	0.021 ± 0.001	0.0029 ± 0.0001	0.032 ± 0.003	0.020 ± 0.001
Gap_T	0.32 ± 0.04	0.015 ± 0.001	0.0018 ± 0.0001	0.022 ± 0.001	0.016 ± 0.001
Gap_G	0.25 ± 0.02	0.018 ± 0.001	0.0028 ± 0.0002	0.022 ± 0.01	0.028 ± 0.002
Gap_C	0.34 ± 0.02	0.023 ± 0.001	0.0041 ± 0.0001	0.023 ± 0.001	0.016 ± 0.002

* Data from ref. [26].

**Table 6:** The rate constant of the chemical step (*k*_pol_) and apparent dissociation constant *K*_d,app(dATP)_.

	WT *	E154A	G189D	M236T	R254I
*k*_pol_, s^−1^	0.93 ± 0.05	1.2 ± 0.2	0.22 ± 0.03	0.89 ± 0.04	0.9 ± 0.2
*K*_d,app(dATP)_, μM	16 ± 3	130 ± 40	230± 70	43 ± 6	120 ± 60

* Data from ref. [26].

## Data Availability

Raw experimental data are available from O.A.K. and A.A.K. upon request.
